# Supplementary material for: Association between oral microbiome and seven types of cancers in East Asian population: a two-sample Mendelian randomization analysis
Source: Front Mol Biosci. 2023 Nov 21;10:1327893. doi: 10.3389/fmolb.2023.1327893 (PMC10702768; doi:10.3389/fmolb.2023.1327893)
Supplement: Supplementary file 1 [file DataSheet1.ZIP › Supplementary Materials/MR plots of saliva/saliva═╝/ovarian cancer/pheno.2070_to_ovarian cancer_leave_one_out.pdf]

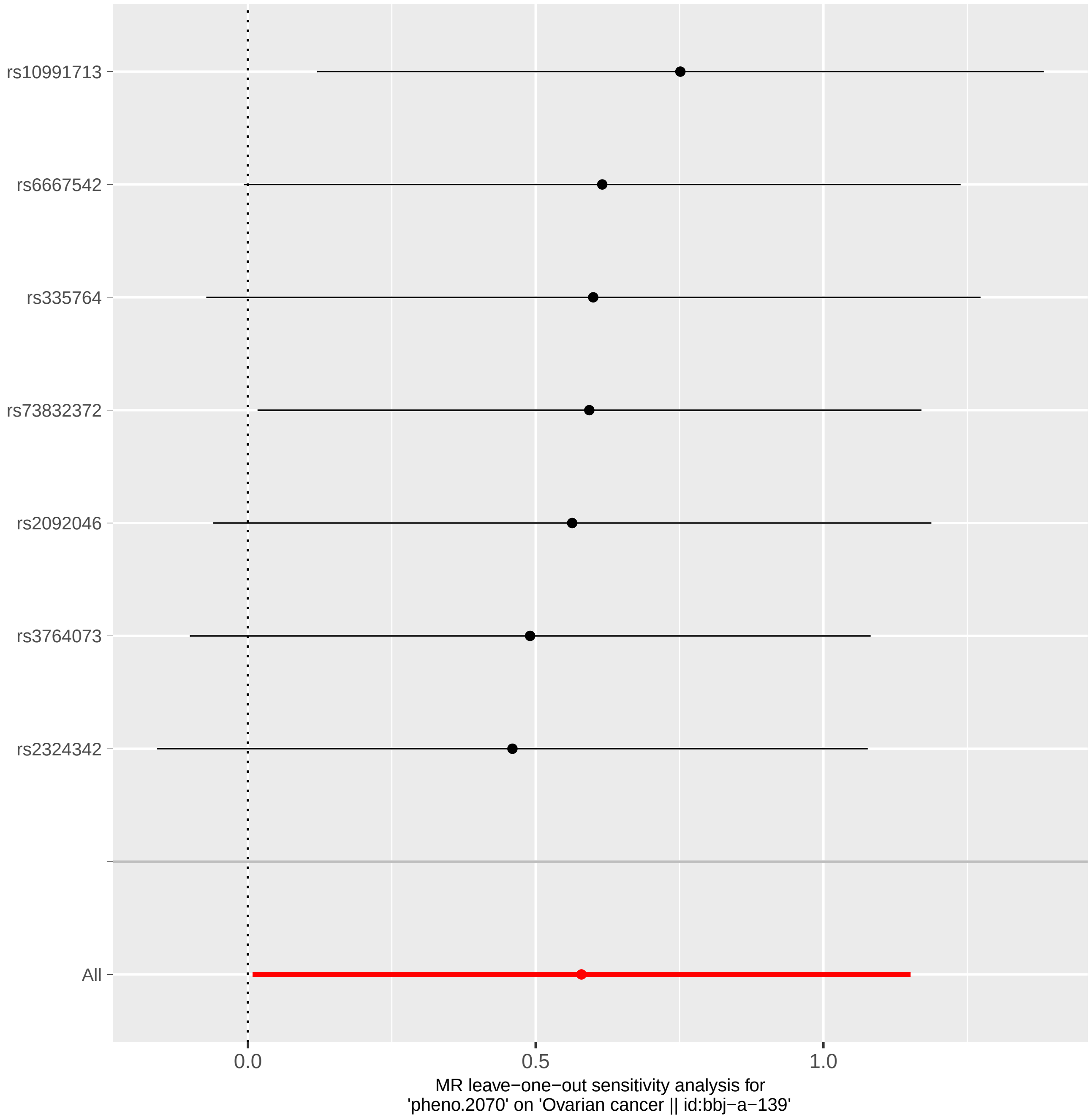
rs10991713
rs6667542
rs335764
rs73832372
rs2092046
rs3764073
rs2324342
All
0.0
0.5
1.0
MR leave−one−out sensitivity analysis for
'pheno.2070' on 'Ovarian cancer || id:bbj−a−139'
